# Bioactive Potential of Sweet Cherry (*Prunus avium* L.) Waste: Antioxidant and Anti-Inflammatory Properties for Sustainable Applications

**DOI:** 10.3390/foods14091523

**Published:** 2025-04-26

**Authors:** Luisa Frusciante, Collins Nyaberi Nyong’a, Alfonso Trezza, Behnaz Shabab, Tommaso Olmastroni, Roberta Barletta, Pierfrancesco Mastroeni, Anna Visibelli, Maurizio Orlandini, Luisa Raucci, Michela Geminiani, Annalisa Santucci

**Affiliations:** 1 Department of Biotechnology, Chemistry and Pharmacy, University of Siena, Via Aldo Moro, 53100 Siena, Italy; luisa.frusciante@unisi.it (L.F.); c.nyonga@student.unisi.it (C.N.N.); alfonso.trezza2@unisi.it (A.T.); b.shabab@student.unisi.it (B.S.); tommaso.olmastroni@student.unisi.it (T.O.); r.barletta@student.unisi.it (R.B.); p.mastroeni@student.unisi.it (P.M.); anna.visibelli2@unisi.it (A.V.); maurizio.orlandini@unisi.it (M.O.); luisa.raucci@unisi.it (L.R.); annalisa.santucci@unisi.it (A.S.); 2 SienabioACTIVE, University of Siena, Via Aldo Moro, 53100 Siena, Italy; 3 ARTES 4.0, Viale Rinaldo Piaggio, 34, 56025 Pontedera, Italy

**Keywords:** circular bioeconomy, *Prunus avium*, sweet cherry, RAW 264.7, antioxidant, anti-inflammatory, DNA damage prevention, MAPK, NF-κB, molecular docking

## Abstract

This study presents an innovative approach to the sustainable valorization of industrial sweet cherry (*Prunus avium* L.) waste from the Vignola Region, Italy, transforming what is typically discarded into a high-value bioactive resource. Unlike conventional extractions, our hydroethanolic extract (VCE) was obtained from the entire cherry waste, including the pericarp, pulp, and stone, as generated by industrial processing. This full-fruit extraction strategy represents a novel and efficient use of agricultural by-products, aligning with circular bioeconomy principles. Sweet cherries are known for their phenolic richness, and spectrophotometric assays (TPC, TFC, reducing power, DPPH, and ABTS) confirmed the extract’s antioxidant capacity. In vitro studies using RAW 264.7 macrophages revealed no cytotoxic effects (MTT assay), along with significant anti-inflammatory activity, evidenced by reduced ROS and NO production and downregulation of iNOS and COX-2. Western blotting showed inhibition of NF-κB nuclear translocation and MAPK pathway signaling. Additionally, agarose gel electrophoresis showed protection against oxidative DNA damage. UPLC-MS/MS analysis identified sakuranetin, aequinetin, and dihydrowogonin as the most representative compounds in VCE. Molecular docking simulations revealed strong and specific binding affinities of these compounds to NF-κB p65 and key MAPK targets. These findings highlight whole sweet cherry waste—including the pit—as a potent and sustainable source of bioactive compounds with promising nutraceutical and pharmaceutical applications.

## 1. Introduction

As sustainability becomes a central global priority, there is an increasing global pursuit of sustainability, driven by a transformative approach to resource management that aims to change traditional paradigms of agricultural waste management. In recent years, numerous research efforts have focused on integrating bioeconomy and biomedicine. At the core of this transformative approach is the concept of a “circular bioeconomy”, which primarily focuses on maximizing resource efficiency, reducing waste, and sustainably promoting bio-based resources [1]. A driving force in this emerging field is a paradigm shift that seeks to transform agricultural and food industry waste into valuable resources for the biomedicine sector. Increasing attention is being given to the use of underutilized agricultural products and food industry by-products. However, the challenges surrounding food industry waste are complex, and further research is necessary to fully harness the industrial potential of these materials as cost-effective and sustainable alternatives for various applications [2].

Europe’s commitment to combating climate change is reflected in its bioeconomy initiatives, which center on the circular bioeconomy, a renewable system that balances necessities such as food, land, materials, and health with the natural world [3]. This means that biodiversity is key to achieving circularity. The goal of the new bioeconomy vision is to transform organic waste, such as crop residues, industrial by-products, and food waste, which the FAO estimates comprise one-third of global food production [4], into value-added products, while also utilizing residual biomass. Biorefinery technologies enable the more effective use of biological resources. The increased valuation of previously overlooked or undervalued plant and animal parts, including aquatic (freshwater and marine) resources, is another feature of the new bioeconomy [3,5]. Notably, the resulting portfolio of bio-based products comprises a wide range of value-added goods that meet various societal and consumer needs.

In light of the growing need for safer and more effective treatments for modern chronic diseases, often associated with lifestyle and aging, and the abundance of bioactive compounds found in plants, especially in plant waste and by-products with notable biological activities and health benefits, the sweet cherry (*Prunus avium* L.), one of the most widely consumed fruits globally, has gained attention. Many countries produce sweet cherries, and both the primary fruit and the by-products generated during industrial processing, including the entire fruit waste, have shown great potential as sources for developing high-value bioactive products [6]. Between 2008 and 2018, global sweet cherry production grew from 1.8 to 2.5 million tons. Europe accounts for around 40% of this output, with China also serving as a major producer. Due to the fruit’s limited shelf life and seasonal availability, roughly 40% of sweet cherries are processed, resulting in considerable amounts of by-products such as stems, seeds, and pomace [7]. By 2020, worldwide cherry pit generation was estimated at nearly four million tons, with the United States alone producing approximately 400,000 tons annually. Remarkably, over 99% of cherry processing in the U.S. takes place in the Great Lakes region, where cherry pit waste is heavily concentrated [8].

There are numerous cherry species within the genus *Prunus*, with hundreds of varieties depending on the cultivar and region [6]. *P. avium* is a deciduous tree that originated in Asia and belongs to the Rosaceae family, subfamily *Prunoideae*, genus *Prunus*, and subspecies *avium*. Commercial cherries are derived from various species, including the sour *P. cerasus* and the sweet *P. avium* [9]. Backed by scientific research, the global consumption and economic value of sweet cherries is on the rise, primarily driven by their potential therapeutic benefits. Sweet cherries have been the subject of extensive scientific research. There is substantial evidence documenting the bioactive compounds present in all parts of the sweet cherry, including by-products such as the skin, pits, leaves, flowers, and other residues often discarded after sorting [10]. The exocarp, or skin, the outermost layer of the fruit, is especially rich in anthocyanins, the pigments responsible for the red, purple, or black coloration of sweet cherries [11,12]. The mesocarp, or fleshy part beneath the skin, contains high levels of vitamin C, which supports immune function and has antioxidant properties, along with natural sugars such as fructose and glucose that contribute to the fruit’s sweetness [13]. The endocarp, the hard layer surrounding the seed, is part of the pit, which, along with other processing by-products, has attracted increasing attention for its potential value. Cherry pomace, made primarily from the skins and pulp, is a notable source of phenolic compounds, including phenolic acids, flavonols, and anthocyanins. These are mainly concentrated in the skin and can make up over 70% of the total phenolic content, positioning pomace as a promising ingredient for natural antioxidants, nutraceuticals, and colorants [7]. Stems, traditionally used for their diuretic and anti-inflammatory effects, have shown in vitro anti-inflammatory activity through the inhibition of inflammatory marker production in macrophages [14]. Seeds, which are rich in oils containing essential fatty acids and other beneficial bioactive compounds, are widely used in cosmetic and pharmaceutical applications [15]. Moreover, extracts from leaves and flowers have demonstrated both anti-inflammatory and antimicrobial activity against various bacterial strains, further supporting the therapeutic potential of cherry by-products [14]. Despite the documented benefits of sweet cherry components, there is limited research on the sustainable management of the substantial amount of waste generated during the sorting of cherries for quality. In a world striving to make better use of its resources, the concept of waste in the agricultural and food sectors challenges the principles of a circular bioeconomy, offering a significant opportunity for further exploration.

This study investigates the sustainable and bioeconomic potential of waste derived from *P. avium*, specifically sweet cherries cultivated in the Vignola region of Italy, which hold the “Vignola Protected Geographical Indication (PGI)” certification. Renowned as the cherry capital, Vignola is known for its prized varieties, including Moretta di Vignola, Nero di Vignola, Duroni, and Nellone [16]. The research focuses on evaluating the antioxidant and anti-inflammatory properties of a hydroethanolic extract obtained from the whole fruit waste. By analyzing its effects on oxidative stress and inflammation, the study aims to highlight the extract’s potential as a source of bioactive compounds with therapeutic value. Ultimately, this work contributes to the advancement of sustainable bioeconomic practices by promoting the use of agricultural by-products in health-related applications, fostering both environmental sustainability and economic resilience.

## 2. Materials and Methods

### 2.1. Materials

Absolute ethanol (HPLC grade), acetonitrile (HPLC grade), formic acid, Dulbecco’s Modified Eagle’s Medium (DMEM), and all the solvents used for cell culture were purchased from Merck (Darmstadt, Germany). RAW 264.7 cells were from ATCC (Manassas, VA, USA). All reagents and materials used were within their validity period.

### 2.2. Preparation of Vignola Cherry Ethanolic (VCE) Extract

Post-industrial sweet cherry waste from *P. avium* L., bearing the Vignola Protected Geographical Indication (PGI) certification, was collected following industrial sorting. The waste included whole non-compliant cherries comprising the pericarp (exocarp, mesocarp, endocarp), pit, and peduncle.

The plant material was first freeze-dried and then pulverized to a fine powder using a laboratory grinder. A total of 10 g of the powdered material was subjected to heat-reflux extraction in 100 mL of ethanol/water (70:30 *v*/*v*) at 80 °C under constant stirring for 3 h. After extraction, the mixture was centrifuged and filtered, and the solvent was removed using a rotary evaporator. The resulting extracts was then freeze-dried to obtain the Vignola cherry extract (VCE). The VCE samples were stored in amber glass vials at −20 °C, protected from light and humidity to minimize degradation of thermolabile and light-sensitive compounds. All analyses were conducted within two weeks of extraction. The extraction was performed in technical triplicate from the same bulk batch of raw material. The extraction yield was calculated as the mean ± standard deviation (SD) and expressed as a percentage of the dry weight of the starting material. The efficiency and reproducibility of the extraction process were assessed through antioxidant activity measurements. All antioxidant assays, including total phenolic content (TPC), total flavonoid content (TFC), total reducing power (TRP), and free-radical scavenging activity (DPPH and ABTS), were conducted in technical triplicate for each extract.

### 2.3. Total Phenolic Content (TPC)

The TPC was quantified by the Folin–Ciocalteu (FC) method as described before [17]. Briefly, a calibration curve was prepared using gallic acid (GA) solutions ranging from 20–120 μg/mL. VCE samples were diluted from a 2 mg/mL stock in Milli-Q water. Standards and samples were mixed with 1 mL of 1N FC reagent, followed after 3 min by 1 mL of saturated Na_2_CO_3_ and 7 mL of Milli-Q water. After 90 min of incubation at room temperature in the dark, absorbance was measured at 725 nm. A blank containing all reagents without sample was also prepared. TPC was reported as milligrams of GA equivalents (GAE) per gram of dry extract.

### 2.4. Total Flavonoid Content (TFC)

The TFC was determined using a modified NaNO_2_–Al(NO_3_)_3_–NaOH colorimetric method based on the procedure by [18]. Briefly, 1 mL of extract was mixed with 0.3 mL of 5% NaNO_2_ in water. After 5 min, 0.3 mL of 10% Al(NO_3_)_3_ was added, followed by a 6 min reaction period. Then, 4 mL of 4% NaOH was added, and the final volume was adjusted to 10 mL with water. After incubation at room temperature for 6 min, absorbance was measured at 510 nm. Blanks were prepared, replacing Al(NO_3_)_3_ and NaOH with equal volumes of water. Standard solutions of rutin were treated similarly to construct a calibration curve ranging from 25 to 200 µg/mL. Flavonoid content was expressed as mg rutin equivalents (RE) per g of dry extract.

### 2.5. Determination of Reducing Power

The total reducing power (TRP) of VCE extracts was evaluated using the potassium ferricyanide reducing power assay as previously described [19]. A calibration curve was prepared using ascorbic acid (20–140 μg/mL). VCE samples (from 2 mg/mL stock) and a water blank were mixed with 1 mL of 0.2 M phosphate buffer (pH 6.6) and 1 mL of 1% potassium ferricyanide, then incubated at 50 °C for 20 min. After the addition of 1 mL of 10% trichloroacetic acid and a resting period of 10 min at room temperature, 2.5 mL of water and 0.5 mL of 0.1% ferric chloride were added. Absorbance was read at 700 nm. TRP was expressed as mg ascorbic acid equivalents (AAE) per g dry extract.

### 2.6. ABTS^+^ Free-Radical Scavenging Activity

Trolox equivalent antioxidant capacity (TEAC) assay measures the ability of compounds to reduce ABTS^+^ radicals to ABTS by molecules able to neutralize the radical [20]. The assay was performed using the OxiSelect™ TEAC Assay Kit (Cell Biolabs Inc., San Diego, CA, USA) according to the manufacturer’s instructions. Briefly, 25 µL of sample at varying concentrations was added to 150 µL of freshly diluted ABTS + reagent (1:50) in a 96-well plate. After 5 min of shaking, absorbance was recorded at 405 nm. Results were expressed as mg Trolox equivalents (TE)/g dry extract.

### 2.7. DPPH Free-Radical Scavenging Activity

DPPH free-radical scavenging activity was determined by measuring the reduction of the stable DPPH (2,2-diphenyl-1-picrylhydrazyl) radical, as previously described [21]. Briefly, samples were mixed with 100 µM DPPH solution and incubated in the dark at 37 °C for 30 min. Absorbance was measured at 517 nm, with a decrease indicating antioxidant activity. A Trolox standard curve was used, and results were expressed as mg TE/g.

### 2.8. UHPLC-ESI-MS/MS

UHPLC-ESI-MS/MS analysis was performed on the selected extract. VCE was resuspended in 1 mL of 75% ethanol, briefly vortexed, and filtered through a 0.2 μm membrane filter. One milliliter of the filtrate was transferred to a UPLC vial for analysis. The analysis was performed using a UHPLC Ultimate 3000 System coupled with a Q Exactive Plus mass spectrometer (Thermo Fisher Scientific, Waltham, MA, USA) equipped with an electrospray ionization (ESI) source. Chromatographic separation was conducted on an Acquity UPLC BEH C18 column (1.7 μm, 2.1 × 150 mm, Waters) maintained at 35 °C, with a 10 μL injection volume. The mobile phases were 0.1% formic acid in water (phase A) and 0.1% formic acid in acetonitrile (phase B), using a linear gradient from 40% B to 100% B over 50 min at a flow rate of 200 μL/min. Mass spectrometric data were acquired in data-dependent acquisition (DDA) mode, selecting the top 10 precursor ions in both positive and negative ESI modes. Data processing and metabolite identification were performed using Compound Discoverer 3.3 (Thermo Fisher Scientific), with comparisons to established metabolite databases. Additional data filtering and analysis were carried out using a custom in-house algorithm and a proprietary, sample-specific database. All instruments were properly calibrated and validated before use.

### 2.9. In Vitro Anti-Inflammatory Activity on RAW 264.7 Cells

#### 2.9.1. Cell Cultures

RAW 264.7 macrophage cells (ATCC, Manassas, VA, USA) were cultured in DMEM supplemented with 10% Fetal Bovine Serum, 100 mg/mL penicillin, and 100 mg/mL streptomycin. Cells were maintained at 37 °C in a humidified 5% CO_2_ atmosphere. Comparative analyses were performed using cells from the same generation.

#### 2.9.2. Cell Viability

RAW 264.7 cells were seeded at 1 × 10^4^ cells/well in 96-well plates and cultured to 80–85% confluence. They were then treated with VCE at concentrations of 50, 100, 200, 400, and 800 µg/mL, dissolved in DMSO (Sigma-Aldrich) and diluted in medium, keeping the final DMSO concentration below 0.8% *v*/*v*. Control wells received 0.8% *v/v* DMSO, matching the highest VCE concentration. Cell viability was assessed using the Cell Counting Kit-8 (CCK-8, Sigma-Aldrich, St. Louis, MO, USA) according to the manufacturer’s instructions. Absorbance was read at 450 nm using a CLARIOstar microplate reader (BMG Labtech, Ortenberg, Germany), and viability was expressed as a percentage relative to the vehicle control. The assay was performed in biological triplicate, using independently cultured RAW 264.7 cell batches. Each biological replicate included technical triplicates.

#### 2.9.3. Cell Stimulation

Cells were pre-treated with VCE prior to stimulation with lipopolysaccharide (LPS) from Escherichia coli O111:B4 (Sigma-Aldrich). Dexamethasone (DEX), a reference anti-inflammatory agent, was included as a positive control at 5 µg/mL (Sigma-Aldrich).

#### 2.9.4. Quantification of Intracellular ROS Generation

The generation of reactive oxygen species (ROS) in RAW 264.7 cells was assessed using 2′,7′-dichlorodihydrofluorescein diacetate (DCFH_2_-DA) as previously described [22]. Briefly, cells were pre-treated with VCE at concentrations of 200, 400, and 800 µg/mL, followed by stimulation with LPS (200 ng/mL) for 5 h. Subsequently, 10 µM DCFH_2_-DA in Hank’s Balanced Salt Solution was added, and cells were incubated for 10 min at 37 °C. Fluorescence was measured at 485/535 nm (excitation/emission) using an EnVision system (PerkinElmer). To normalize for cell number, a crystal violet assay was performed, staining the cells with 0.1% crystal violet, followed by ethanol solubilization and absorbance measurement at 570 nm. ROS production was expressed as the percentage relative to the LPS-treated control group. Quantification of intracellular ROS production was conducted in biological triplicate, with three independent experiments carried out on different days. Each experiment was performed in technical triplicate.

#### 2.9.5. Determination of NO Production

Nitric oxide (NO) production in the supernatant of RAW 264.7 cells was measured in 6-well plates seeded at 1 × 10^6^ cells/well and cultured to 80–85% confluence. Cells were treated with VCE (25, 50, and 100 μg/mL) for 4 h, followed by stimulation with LPS (200 ng/mL) for 24 h. Then, 100 µL of the conditioned medium was transferred to a 96-well plate and mixed with 100 µL of Griess reagent (1% sulfanilamide and 0.1% N-(1-naphthyl)ethylenediamine dihydrochloride in 5% phosphoric acid). After a 10 min incubation at room temperature, absorbance was read at 540 nm using an EnVision system (PerkinElmer). Nitrite levels were determined using a sodium nitrite standard curve. Determination of NO production was conducted in biological triplicate, with three independent experiments, each performed in technical triplicate.

#### 2.9.6. Protein Extraction

Whole-cell lysates were obtained using RIPA buffer containing phosphate and protease inhibitors, followed by 15 min of sonication in an ice bath for cell disruption. Protein concentration was measured using the bicinchoninic acid (BCA) assay. For nuclear fractionation, the NE-PER™ Cytoplasmic and Nuclear Protein Extraction Kit (Thermo Fisher Scientific, Rockford, IL, USA) was used according to the manufacturer’s instructions.

#### 2.9.7. Western Blotting

20 μg of protein were resolved by SDS–PAGE and transferred onto a nitrocellulose membrane. The membrane was blocked in TBS 5% *w/v* nonfat dry milk at RT with gentle shaking for 2 h. The membrane was incubated with anti-iNOS (rabbit polyclonal IgG, 1:10,000 Sigma-Aldrich), anti-COX-2 (rabbit polyclonal IgG, 1:4000 Sigma-Aldrich), anti-NF-κB p65 (mouse monoclonal clone 1G10.2, 1:1000 Sigma-Aldrich), anti-Nucleolin (rabbit polyclonal, 1:10,000 Sigma-Aldrich), anti-pJNK and anti-JNK (rabbit polyclonals, 1:1000 Sigma-Aldrich), anti-ERK (1:20,000 Sigma-Aldrich), and p-ERK (1:2000 Cell Signaling) primary antibodies and anti-β-actin (1:5000 Sigma Aldrich) secondary antibody, ON at 4 °C. The blots were washed three times and incubated with anti-rabbit HRP-conjugated secondary antibody (Sigma-Aldrich) 1:80,000 or anti-mouse HRP-conjugated secondary antibody (Sigma-Aldrich) 1:50,000 for 1 h, RT. After three washes, immunoreactive bands were detected using ECL (LuminataCrescendo, Merck Millipore, Burlington, MA, USA) and images acquired by LAS4000 (GE Healthcare, Chicago, IL, USA). The optical densities of immunoreactive bands were analyzed by ImageQuantTL software (GE Healthcare, Chicago, IL, USA, V 7.0) using β-actin, ERK, JNK, or Nucleolin as loading normalizing factors. Protein expression analysis by Western blot was performed in biological triplicate, with protein extracted from three independent cell cultures. Densitometry was conducted on replicate blots.

#### 2.9.8. Immunofluorescence Study

RAW 264.7 cells were cultured on glass coverslips for 24 h, then pre-treated with VCE (100 μg/mL) for 4 h, followed by LPS stimulation for 1 h. Cells were fixed with 4% paraformaldehyde in PBS for 15 min, washed three times, and permeabilized with 0.5% Triton X-100 in PBS for 5 min. After blocking with 5% Normal Goat Serum (NGS) in PBS for 20 min, cells were incubated overnight at 4 °C with anti-NF-κB p65 mouse monoclonal antibody (1:200, Sigma-Aldrich). Following three PBS washes (10 min each), cells were incubated for 1 h in the dark at room temperature with Alexa 594-conjugated goat anti-mouse IgG (1:100, Life Technologies, Carlsbad, CA, USA). After three PBS washes and one with distilled water, cells were mounted with fluoroshield containing DAPI. Images were captured using a Zeiss AxioLabA1 fluorescence microscope (Oberkochen, Germany).

### 2.10. Antimutagenicity in DNA Oxidative Damage

A study was conducted to evaluate the protective effect of VCE against Fenton-induced DNA damage using supercoiled pET22b plasmid, following the method described by [23] with minor modifications. Briefly, 500 ng of plasmid DNA was pre-treated with VCE at concentrations of 200 and 400 µg/mL for 10 min at room temperature. Simultaneously, a Fenton reaction mixture was prepared, combining freshly prepared 0.25 mM FeSO_4_ and 2.5 mM H_2_O_2_. After the pre-treatment period, the Fenton mixture was added to the DNA samples and incubated at 37 °C for 30 min. Trolox (6-hydroxy-2,5,7,8-tetramethylchromane-2-carboxylic acid; Sigma) at a concentration of 100 µg/mL was used as a positive control, while a negative control containing DNA alone was also included. Following incubation, samples were loaded onto a 0.8% agarose gel stained with Eurosafe (Euroclone) and subjected to electrophoresis in 1 × Tris-Acetate-EDTA (TAE) buffer at 90 V for 30 min. DNA integrity was assessed, and images were captured using the LAS4000 imaging system (GE Healthcare, Chicago, IL, USA). The assay was conducted in biological triplicate, using extracts in three independent experiments.

### 2.11. Statistical Analysis

Experiments were performed in triplicate. Statistical analyses were performed with GraphPad Prism 9.0 software (GraphPad Software, San Diego, CA, USA). Data are presented as mean ± SD and were compared using the unpaired *t*-test or the one-way ANOVA with an appropriate post hoc test. A *p* value of 0.05 or less was considered significant.

### 2.12. In Silico Studies

Structural Resources and docking simulation.

The primary structures of human NF-κB p65, c-Jun N-terminal kinase 3 (MAPK10), and ERK1 (MAPK3) were obtained from UniProtKB with entries “Q04206”, “P53779”, and “P27361”, respectively [24]. The 3D structures for NF-κB p65 and human c-Jun N-terminal kinase 3 (MAPK10) were retrieved from the RCSB Protein Data Bank [25] with PDB codes “1NFI” and “1PMN”, respectively. The 3D structure of ERK1 (MAPK3) was generated by AlphaFold [26].

Potential missing side chains and steric clashes were adjusted through molecular modelling PyMOD3.0 [27] and validated with PROCHECK v.3.5.4 [28].

The 3D structures of the three most abundant compounds of the extract were used for the docking simulation. Due to the lacking bioactivity of the glycosylated molecule sakuranin, its aglycone, sakuratenin, was instead used for these studies [29]. Sakuratenin (CID: 73571), aequinetin (CID: 15558425) and dihydrowogonin (CID: 11491431) were sourced in sdf format from the PubChem database [30].

Docking simulations were carried out, as detailed in previous research [31], to explore the interactions between the above-mentioned molecules and the targets predicted to be involved in the compounds’ biological activity. To enhance the consistency of docking results, the exhaustiveness parameter was increased from 8 to 32, and only binding poses with an RMSD of less than 2 Å from the optimal docked pose were considered acceptable, while all other settings were left as default. A docking box of 20 Å along each dimension was configured around residues known to interact with target inhibitors, utilizing DockingPie2.0 integrated with PyMOL 3.0 [32] and AutoDock Tools v.4.2 [33].

Subsequent docking simulations were carried out using AutoDock/VinaXB v.1.1.2 [34]. Protein and ligand files were prepared and converted using MGLTOOLS v.1.5.7 scripts, and Gasteiger partial charges were applied using OpenBabel v.3.1.0 [35].

Interaction networks were analyzed using the P.L.I.P. v.2.3.0 tool [36,37], while sequence alignments to identify key target residues were performed with ClustalW v.2.1 [38].

## 3. Results

### 3.1. Extraction and Chemical Composition of P. avium Extract

**Figure 1:**
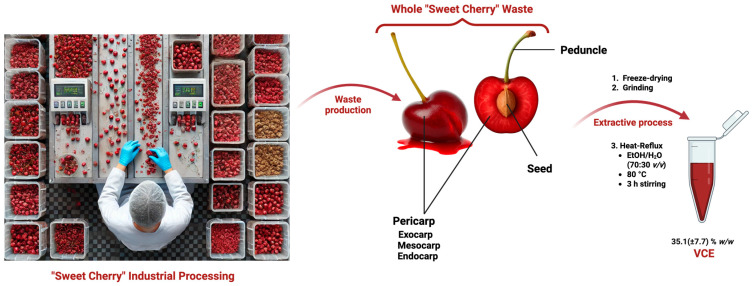
Workflow of VCE production: industrial cherry sorting → whole waste recovery (pericarp, pit and seed, peduncle) and subjection to extraction → heat-reflux extraction (EtOH/H_2_O, 70:30 *v*/*v*) → Vignola cherry extract (VCE).

The ethanolic extract of Vignola cherry waste (VCE), obtained via heat-reflux extraction using an ethanol/water mixture (70:30 *v*/*v*), was first evaluated to assess the efficiency of the extraction process. The waste material included the entire cherry sorting by-product—comprising the pericarp (mesocarp, endocarp, and exocarp), seed, and peduncle—representing a comprehensive valorization approach (Figure 1). The extraction yield was calculated as 35.1 ± 7.7% (*w*/*w*), based on technical triplicates performed using aliquots from the same bulk batch of raw material.

To determine the antioxidant potential of the obtained extracts, TPC and TFC were quantified using colorimetric assays. VCE exhibited a TPC of 14.6 ± 1.3 mg GAE/g of dry extract and a TFC of 24.8 ± 3.4 mg QE/g of dry extract (Table 1).

The reducing power, assessed using the potassium ferricyanide method, was measured at 11.9 ± 1.1 mg AAE/g of dry extract. Free radical scavenging activity was evaluated through ABTS^+^ and DPPH assays, with VCE displaying values of 54.3 ± 6.4 mg TE/g and 16.8 ± 1.6 mg TE/g, respectively.

Further phytochemical characterization of VCE was performed using UHPLC-ESI-MS/MS. A total of 87 metabolites were identified using Compound Discoverer 3.3, supported by the ChemSpider and mzCloud databases, and confirmed through comparison with literature data. The identified compounds are reported in Table 2.

**Figure 2:**
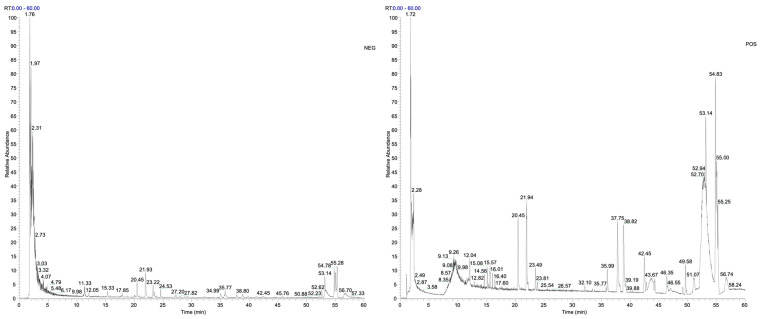
UHPLC-ESI-MS/MS base peak chromatograms of the VCE extract acquired in negative and positive ionization modes over the *m*/*z* range 100–1500.

For each compound, the molecular ion is presented as a combination of *m/z* value and ion type ([M−H]^−^ or [M+H]^+^), and all identifications showed a mass error below 5 ppm. UHPLC-ESI-MS/MS base peak chromatograms acquired in both negative and positive ionization modes are shown in Figure 2.

### 3.2. VCE Reduces LPS-Induced Inflammatory Markers in RAW 264.7 Cells

**Figure 3:**
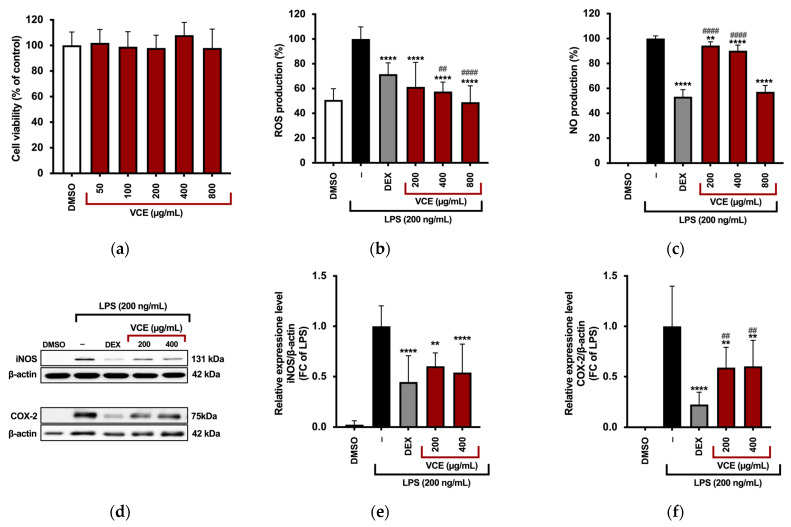
(**a**) Viability of RAW 264.7 cells after 24 h treatment with various concentrations of VCE, assessed by MTT assay. (**b**) Intracellular ROS levels measured following pre-treatment with DEX (5 µg/mL) or VCE (200, 400, and 800 µg/mL), followed by LPS stimulation (200 ng/mL) for 5 h. ROS levels were quantified based on relative fluorescence intensity and normalized to cell number using the crystal violet assay. All experiments were performed in triplicate. Data are presented as bar graphs and expressed as a percentage of control (mean ± SD). Statistical significance was determined by one-way ANOVA with Tukey’s post hoc test. **** *p* < 0.0001 (vs. LPS); ^##^ *p* = 0.0048 and ^####^ *p* < 0.0001 (vs. DEX as positive control). (**c**) Effect of VCE on LPS-induced inflammation in RAW 264.7. Cells were pre-treated with DEX or VCE for 4 h, followed by stimulation with LPS for 24 h. NO production was assessed using the Griess assay, while iNOS (**d**,**e**) and COX-2 (**d**,**f**) expression levels were evaluated by Western blot analysis. β-actin was used as a loading control for normalization of immunoreactive bands. Data are expressed as mean ± SD from three independent experiments. Statistically significant differences are indicated by ** *p* ≤ 0.0045 and **** *p* < 0.0001 (vs. LPS); ^##^ *p* ≤ 0.0099 and ^####^ *p* < 0.0001 (vs. DEX). *p*-values were calculated using one-way ANOVA with Tukey’s post hoc test.

The cytotoxicity of VCE in RAW 264.7 macrophages was evaluated using the MTT assay. As shown in Figure 3a, cell viability was expressed as a percentage relative to control cells treated with DMSO (used as the vehicle at a final concentration not exceeding 0.8% *v*/*v*), which did not affect any of the measured parameters. VCE treatment at concentrations of 50, 100, 200, 400, and 800 µg/mL did not significantly compromise cell viability, indicating a lack of cytotoxicity across the tested range.

Based on these results, concentrations of 200, 400, and 800 µg/mL were selected for subsequent experiments assessing reactive oxygen species (ROS) production. ROS levels were quantified using a DCFH-DA fluorescence assay in LPS-stimulated RAW 264.7 cells. LPS exposure markedly increased fluorescence intensity, indicating elevated intracellular ROS. However, pretreatment with VCE at 200, 400, and 800 µg/mL resulted in a significant, dose-dependent reduction in ROS generation compared to LPS-only treated cells (Figure 3b), demonstrating the antioxidant potential of VCE under inflammatory conditions.

Macrophages are central regulators in the initiation and propagation of inflammatory responses. To assess the anti-inflammatory potential of VCE, LPS-stimulated RAW 264.7 murine macrophages were treated with increasing concentrations of the extract (200, 400, and 800 µg/mL), and the production of key pro-inflammatory mediators was evaluated. As expected, dexamethasone (DEX) at 5 µg/mL, used as a positive control, markedly suppressed NO production. Among the tested VCE concentrations, the highest dose (800 µg/mL) reduced LPS-induced NO release with an effect comparable to that of 5 µg/mL DEX (*p* = 0.06) (Figure 3c).

To investigate the underlying mechanisms, the expression of inducible nitric oxide synthase (iNOS), the enzyme responsible for NO synthesis, was analyzed via Western blot. As shown in Figure 3d,e, VCE treatment at 200 and 400 µg/mL significantly reduced iNOS protein levels compared to LPS-stimulated cells. Cyclooxygenase-2 (COX-2), another key pro-inflammatory enzyme, was also downregulated by VCE at both concentrations (Figure 3d,f). In all three parameters, the inhibitory effects of VCE were comparable to those observed with DEX, as the differences were not statistically significant.

### 3.3. VCE Modulates MAPK and NF-κB Inflammatory Pathways in LPS Stimulated RAW 264.7 Cells

**Figure 4:**
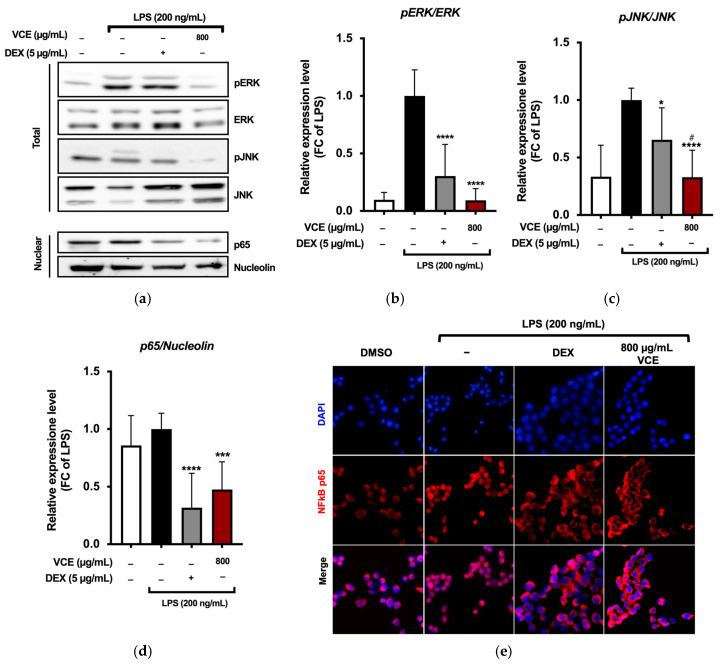
(**a**) VCE treatment suppressed the MAPK and NF-κB signaling pathways in LPS-stimulated RAW 264.7 cells. Cells were pre-treated with various concentrations of VCE for 4 h, followed by LPS stimulation (200 ng/mL) for 1 h. Western blot analysis was used to assess the phosphorylation levels of (**b**) pERK and (**c**) pJNK, as well as (**d**) the nuclear expression of the NF-κB p65 subunit. Band intensities were quantified via densitometry from three independent experiments. Data are expressed as mean ± SD (*n* = 3). * *p* = 0.0177, *** *p* = 0.0003, **** *p* < 0.0001 (vs. LPS); ^#^ *p* = 0.0275 (vs. DEX), determined by one-way ANOVA with Tukey’s post hoc test. (**e**) NF-κB p65 localization was visualized by fluorescence microscopy (red), with nuclei counterstained using DAPI (blue). Images were acquired at 40× magnification.

At a concentration of 800 µg/mL, VCE significantly reduced the phosphorylation of MAPKs in LPS-stimulated RAW 264.7 macrophages, specifically affecting pERK (Figure 4a,b) and pJNK (Figure 4a,c). These inhibitory effects were comparable to those observed with DEX, used as a positive control.

To further elucidate the anti-inflammatory mechanism of VCE, we investigated its influence on the Nuclear Factor kappa-light-chain-enhancer of activated B cells (NF-κB) signaling pathway, a critical regulator of pro-inflammatory gene expression in macrophages [39]. Immunofluorescence analysis revealed that LPS stimulation promoted the translocation of the NF-κB p65 subunit from the cytoplasm to the nucleus, as expected. However, pre-treatment with VCE at 100 µg/mL significantly reduced nuclear localization of p65 (Figure 4d), suggesting inhibition of NF-κB activation. Fluorescence microscopy further confirmed that VCE treatment effectively retained NF-κB p65 in the cytoplasm, preventing its nuclear translocation (Figure 4e).

### 3.4. VCE Protects Plasmidic DNA in Fenton-Induced Oxidation

**Figure 5:**
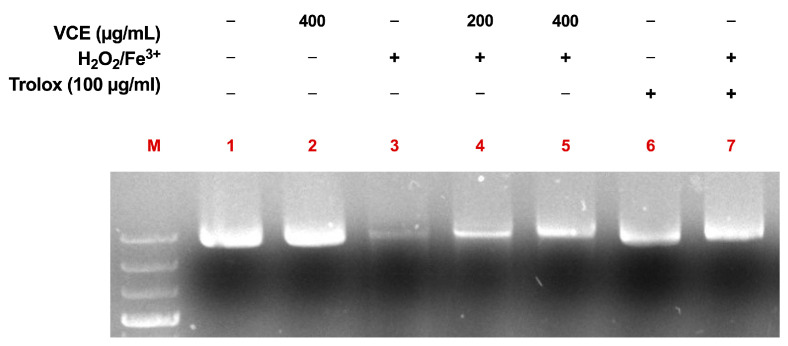
Nicking protection assay on pET22b plasmid DNA. DNA (500 ng) was pre-treated with VCE (200 or 400 µg/mL) for 10 min at RT, then exposed to the Fenton mixture (0.25 mM FeSO_4_ and 2.5 mM H_2_O_2_) at 37 °C for 30 min. Trolox (100 µg/mL) was used as a positive control. Lane 1: untreated DNA; Lane 2: DNA + VCE 400 µg/mL; Lane 3: DNA + H_2_O_2_/Fe^3+^; Lane 4: DNA + VCE 200 µg/mL + H_2_O_2_/Fe^3+^; Lane 5: DNA + VCE 400 µg/mL + H_2_O_2_/Fe^3+^; Lane 6: DNA + Trolox; Lane 7: DNA + Trolox + H_2_O_2_/Fe^3+^.

A DNA nicking assay was conducted to evaluate the protective efficacy of VCE at different concentrations. As shown in Figure 4, Lane 1 (DNA alone) displays intact supercoiled plasmid DNA, serving as the negative control. Lane 2 shows DNA treated with VCE at 400 µg/mL alone, which does not result in any degradation, indicating that the extract itself is not genotoxic. In contrast, Lane 3 (DNA + H_2_O_2_/Fe^3+^) exhibits complete DNA degradation, confirming the damaging effect of hydroxyl radicals generated by the Fenton reaction (Figure 5).

Pre-treatment with VCE at 200 µg/mL (Lane 4) provides partial protection, while a more substantial protective effect is observed at 400 µg/mL (Lane 5), demonstrating a clear dose-dependent response. Lane 6 (DNA + Trolox) confirms that Trolox at 100 µg/mL does not cause DNA damage. Importantly, Lane 7 (DNA + Trolox + H_2_O_2_/Fe^3+^) shows significant protection against oxidative damage, with an effect comparable to that of VCE at 400 µg/mL (Lane 5).

These results suggest that VCE exhibits a concentration-dependent ability to protect DNA from oxidative damage, similar to the well-established antioxidant Trolox.

### 3.5. In Silico Results

Target/compound interactions

Docking simulations were performed for sakuratenin, aequinetin, and dihydrowogonin to NF-κB p65, human c-Jun N-terminal kinase 3 (MAPK10), and ERK1 (MAPK3), targeting a known binding site for their human inhibitors [40]. Two strategies were employed to select the top complexes: (i) selecting compounds with a binding free energy (docking score) lower than −5 kcal/mol and (ii) assessing the compound’s ability to form strong interactions with consensus binding residues of the target.

Docking studies demonstrated that sakuratenin, aequinetin, and dihydrowogonin consistently bound to the target binding pockets across all studied proteins. The binding poses were similar, the strong binding affinities indicated by favorable energy scores. Interaction analysis revealed an extensive network of polar and hydrophobic interactions with key residues in the targets’ sensing regions, supporting the compounds’ potential inhibitory activity [40,41,42,43].

**Figure 6:**
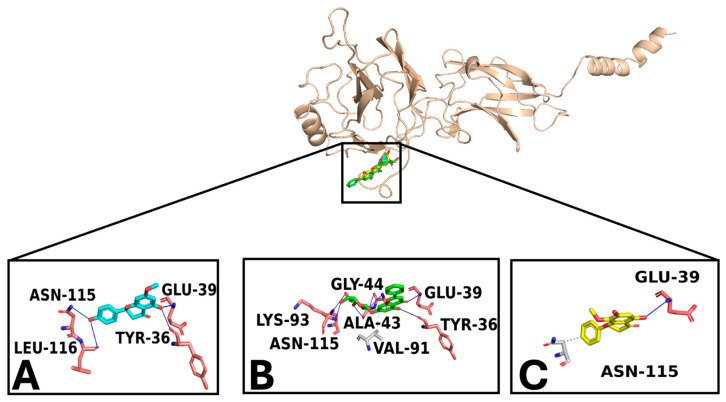
Overview of the NF-kB p65/ligands complexes. (**A**) is sakuratenin; (**B**) is aequinetin, and (**C**) is dihydrowogonin. The zoomed-in pictures display the interaction network among key target binding residues and the ligands. The binding residues involved in hydrogen bonds and hydrophobic interactions are labeled pink and grey, respectively. Hydrogen bonds and hydrophobic interactions are pictured as blue and light grey lines and dashed lines, respectively.

NF-κB p65/compound docking results (Figure 6) showed the ability of sakuratenin (−6.1 kcal/mol), aequinetin (−6.8 kcal/mol), and dihydrowogonin (−5.8 kcal/mol) to interact in a strong way with the target, exhibiting high energy scores and forming a wide interaction network. Interestingly, all compounds docked in a target binding pocket proposed to be an inhibitor binding site of NF-κB p65 [40] and triggered strong polar and hydrophobic interactions with protein-sensing residues, suggesting the potential inhibitory activity of compounds on the target. Specifically, all compounds conserved the establishment of the hydrogen bond with Glu-39, as well as the hydrophobic interaction with Asn-115.

**Figure 7:**
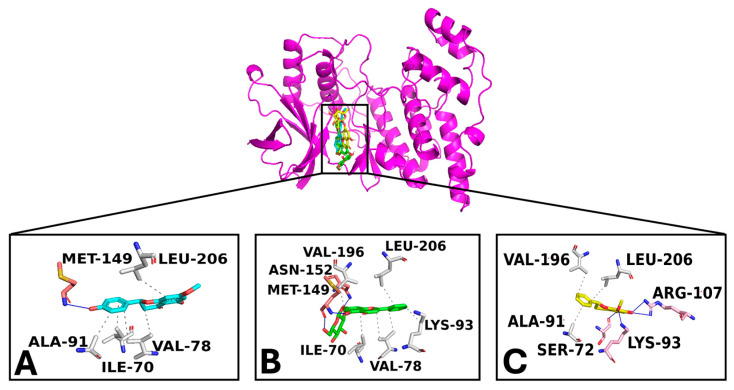
Overview of the MAPK10/ligands complexes. (**A**) is sakuratenin; (**B**) is aequinetin, and (**C**) is dihydrowogonin. The zoomed-in pictures display the interaction network among key target binding residues and the ligands. The binding residues involved in hydrogen bonds and hydrophobic interactions are labeled pink and grey, respectively. Hydrogen bonds and hydrophobic interactions are pictured as blue and light grey lines and dashed lines, respectively.

Docking results for MAPK10 and the compounds sakuratenin (−7.5 kcal/mol), aequinetin (−8.4 kcal/mol), and dihydrowogonin (−7.6 kcal/mol) revealed their strong interaction with this target, displaying high energy scores and a broad polar and hydrophobic interaction network (Figure 7). Notably, all compounds docked into a binding pocket proposed as the inhibitor binding site of MAPK10 [40]. The established interactions included strong contacts with key protein residues, suggesting potential inhibitory activity. Specifically, the given compounds formed hydrogen bonds with Lys-93 and Met-149 and hydrophobic interactions with Ile-70 and Leu-206.

**Figure 8:**
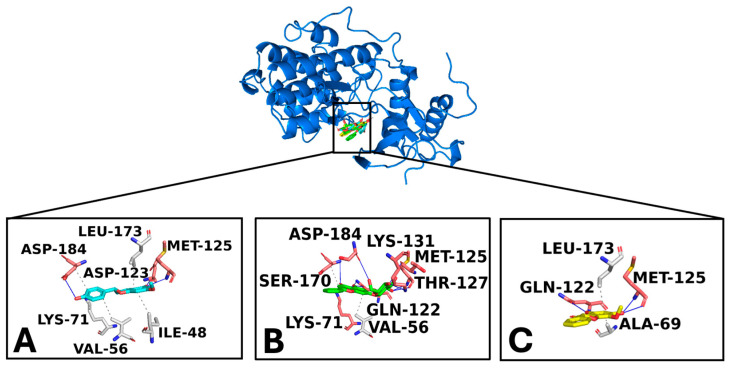
Overview of the ERK/ligands complexes. (**A**) is sakuratenin; (**B**) is aequinetin, and (**C**) is dihydrowogonin. The zoomed-in pictures display the interaction network among key target binding residues and the ligands. The zoomed-in pictures display the interaction network among key target binding residues and the ligands. The binding residues involved in hydrogen bonds and hydrophobic interactions are labeled pink and grey, respectively. Hydrogen bonds and hydrophobic interactions are pictured as blue and light grey lines and dashed lines, respectively.

The docking analysis of ERK demonstrated the robust interaction of sakuratenin (−7.8 kcal/mol), aequinetin (−7.9 kcal/mol), and dihydrowogonin (−8.0 kcal/mol) with the target, supported by favorable energy scores and a network of hydrophobic and polar interactions (Figure 8). Importantly, all three compounds docked within a binding pocket identified as the proposed inhibitor site of a known ERK inhibitor [42]. The preserved interactions included significant contacts with critical protein residues, indicating potential inhibitory effects as the compounds established hydrogen bonds with Met-125 and Ser-170 and hydrophobic interactions with Leu-173.

## 4. Discussion

In an era defined by environmental urgency and increasing health challenges, the convergence of sustainability, waste valorization, and human well-being has never been more critical. The circular bioeconomy offers a compelling solution, transforming agricultural by-products once considered waste into valuable resources that support both ecological balance and therapeutic innovation. This paradigm not only reduces environmental impact but also unlocks the potential of natural compounds to address chronic health conditions. Sweet cherry waste, rich in bioactive molecules, stands as a promising example of how sustainability and health can align through scientific research and innovation.

Plant-based natural remedies represent one of the most ancient and enduring forms of medicine known to humankind. Their adaptability and the diversity of bioactive compounds found in nature have maintained their relevance even in modern medicine. Natural compounds have long played a crucial role in traditional healing practices, and the vast biodiversity of our planet offers a rich reservoir of molecules with therapeutic potential [44,45,46,47,48,49]. In recent years, increasing attention has been directed toward discovering safer and more effective alternatives to synthetic anti-inflammatory drugs, which are often associated with significant side effects and limitations [50].

Chronic inflammatory diseases such as inflammatory bowel disease, cardiovascular disorders, and neurodegenerative conditions pose a growing public health challenge. Inflammation and oxidative stress are now recognized as key contributors to the pathogenesis of these diseases. Although corticosteroids and NSAIDs remain standard treatments, their adverse effects and long-term safety concerns highlight the demand for safer alternatives [51]. The search for new anti-inflammatory agents has turned increasingly to natural products. Derived from plants, animals, and microorganisms, these compounds have historically served as valuable leads for drug development [46,52,53]. Sweet *P. avium* cherries is a promising source of natural bioactive compounds, including flavonoids and phenolic acids, known for their antioxidant and anti-inflammatory properties [54,55]. While the traditional medicinal use of cherry bark and leaves is well-documented, limited data exist on the therapeutic properties of whole cherry fruit extracts, especially from post-harvest waste. This work aims to fill that gap by exploring the antioxidant and anti-inflammatory potential of a hydroethanolic extract obtained from Vignola sweet cherry waste, which includes the exocarp, mesocarp, endocarp, seed, and peduncle. The study focuses on the extract’s ability to reduce inflammation in LPS-stimulated RAW 264.7 macrophages, a widely used in vitro model for inflammation.

The therapeutic efficacy of plant extracts is largely influenced by their phytochemical composition. Sweet cherries are rich in phenolic compounds, particularly flavonoids such as anthocyanins, which give the fruit its characteristic red color and have been associated with anti-inflammatory, anti-atherogenic, and vasodilatory effects [56,57]. Studies have shown that the phenolic content of sweet cherries varies depending on cultivar, climate, and growing conditions, highlighting the need for cultivar-specific evaluations [6,58]. Technological advancements in proteomics, genomics, and analytical chemistry have enhanced our understanding of these bioactive compounds and their functions. Średnicka-Tober et al. demonstrated significant variability in antioxidant activity among commercial sweet cherry cultivars, reflecting differences in phenolic profiles [59]. Local varieties thus represent valuable sources of biodiversity and bioactive compounds, contributing to both health and sustainability [55]. Polyphenols, which include flavonols, flavanols, anthocyanins, flavanones, and hydroxycinnamic acids, are the predominant antioxidants in cherries and constitute an essential part of the human diet [60]. These compounds exhibit various biological effects, including free-radical scavenging and modulation of inflammatory pathways.

The extraction method plays a crucial role in the yield and efficacy of bioactive compounds. Solvent choice, temperature, and processing time can significantly influence extract quality [11]. Ethanol–water mixtures are commonly used due to their safety and efficiency in extracting a broad range of phenolic compounds. In compliance with EU regulations (Directive 2009/32/EC), ethanol is recognized as a safe solvent for food and nutraceutical applications. Using a 70:30 ethanol-water solution, the VCE was prepared from freeze-dried, ground cherry waste, yielding a dry extract rich in phenolic compounds.

The extract demonstrated a strong TPC, which aligns well with its observed antioxidant activity. Comparative analysis with other cherry cultivars further supports the potent phytochemical profile of VCE. In one of the few studies involving whole fruit, “Saco” cherry extracts obtained through fractionated high-pressure extraction showed TPC values ranging from 0.6 to 2.5 mg GAE/g dry extract, approximately six times lower than the TPC of VCE, which reached 14.6 mg GAE/g dry extract [61]. Another study focused on different sweet cherry by-products analyzed separately, reporting TPC values ranging from 2.20 to 28.15 mg GAE/g for skins, 1.52 to 2.15 mg GAE/g for pulp, and 3.80 to 9.22 mg GAE/g for seeds, all expressed per gram of dry plant material. In comparison, the TPC of our VCE extract, when adjusted for extraction yield, corresponds to approximately 5.12 mg GAE/g of dry plant, placing it well within or above the ranges observed in these previous studies [62].

UHPLC-ESI-MS/MS analysis of the extract revealed a complex phytochemical profile, predominantly composed of flavonoids (69%), followed by fatty acids and their conjugates (13%), and phenylpropanoids (C6-C3). This high flavonoid content is consistent with previous studies emphasizing the rich phenolic composition of *P. avium* sweet cherry fruits and their by-products [7,44,47,49]. Among the most abundant flavonoids identified were sakuranin, aequinetin, and dihydrowogonin. Sakuranin, a glycosylated derivative of sakuranetin, has been previously detected in cherry fruit, though it accounted for less than 1% of total phenolics [10]. Comprehensive profiling of cherry peduncles has reported up to 26 phenolic compounds, including 19 flavonoids and seven phenolic acid derivatives. Notable flavonoids include sakuranetin, quercetin, catechin, and epicatechin, with subclass representatives such as kaempferol 3-O-rutinoside (flavonol), catechin (flavanol), chrysin-7-O-glucoside (flavone), and sakuranetin-5-O-glucoside (flavanone) [10]. Non-flavonoid phenolics commonly found include p-coumaric acid, p-coumaroylquinic acid, and pyrogallol. Aequinetin (chrysin 7-O-β-D-glucopyranoside) and dihydrowogonin are flavonoids commonly found in various plant species; however, their presence in *P. avium* extracts, especially those derived from whole fruit waste, has been rarely reported. Only a few studies have identified dihydrowogonin in cherry peduncle extracts [63], and comprehensive investigations specifically targeting these compounds in sweet cherry remain limited. Nevertheless, sweet cherries are widely recognized for their rich polyphenolic profile and the well-documented antioxidant and anti-inflammatory properties of these compounds, which function by scavenging free radicals and modulating redox-sensitive signaling pathways [54,55]. The presence of such bioactive flavonoids in VCE underscores its potential as a natural therapeutic agent for oxidative and inflammatory conditions.

Our results demonstrated a significant reduction in NO and ROS production in LPS-stimulated RAW 264.7 macrophages treated with VCE. Additionally, the extract was able to protect plasmid DNA from oxidative damage induced by the Fenton reaction, indicating robust antioxidant activity. These findings are consistent with earlier reports on the radical-scavenging and anti-inflammatory properties of cherry-derived phenolics [64,65,66]. Mechanistically, the extract modulated key signaling pathways involved in inflammation. Western blot analysis revealed inhibition of phosphorylation of MAPKs, specifically pERK and pJNK, which are crucial for the propagation of inflammatory signals. MAPKs, including ERK and JNK, are activated in response to inflammatory stimuli and regulate the expression of various pro-inflammatory cytokines and enzymes. Inhibition of MAPK phosphorylation, as observed in our study, indicates that the extract can disrupt upstream signaling events, providing an additional mechanism for its anti-inflammatory effects [50,67]. Moreover, we observed nuclear retention of NF-κB p65 in RAW 264.7 cells, suggesting that the extract may interfere with NF-κB nuclear translocation and downstream gene transcription. The role of NF-κB in inflammatory diseases is well documented. In resting cells, NF-κB is sequestered in the cytoplasm by IκB proteins. Upon activation, it translocates to the nucleus to promote the transcription of pro-inflammatory genes [68,69]. Our data showing nuclear retention of p65 suggest that the extract may inhibit this translocation, thereby suppressing gene expression associated with inflammation. Similar effects have been observed with other flavonoids, which act at multiple levels of the NF-κB pathway [70]. The presence of flavonoids such as quercetin, kaempferol derivatives, and pelargonidin further supports these findings. Quercetin, for instance, has been shown to inhibit NF-κB activation and reduce oxidative stress in macrophages [71]; kaempferol derivatives, also found in our extract, have been shown to interfere with the activation and nuclear translocation of NF-κB and to inhibit MAPK signaling, supporting our experimental data [50,71].

Importantly, sakuranin, aequinetin, and dihydrowogonin, the three primary compounds in our extract, have been less extensively studied but show promising in silico binding to MAPK and NF-κB, suggesting they may contribute significantly to the observed bioactivities. Their potential roles in modulating inflammation warrant further investigation. The in silico docking studies provided additional support for these observations. Major compounds identified in VCE were computationally tested against MAPK and NF-κB p65, demonstrating strong binding affinities that suggest potential direct interactions with key inflammatory mediators.

The extract’s ability to reduce oxidative stress, as evidenced by decreased ROS and protection against DNA damage, is particularly relevant in the context of chronic inflammation. ROS are known to amplify inflammatory responses and contribute to tissue damage and disease progression [72,73]. The protective effect of the extract suggests that it can counteract ROS-mediated cellular damage, adding another layer to its anti-inflammatory potential. Chronic inflammation, a prolonged, low-grade inflammatory state implicated in numerous diseases, including cardiovascular disorders, diabetes, and cancer [50,74,75], is often associated with the dysregulation of immune responses and persistent activation of signaling pathways such as NF-κB and MAPKs. Our findings indicate that the Vignola cherry extract may offer a multi-targeted approach to modulate these pathways, reduce oxidative stress, and mitigate inflammation.

Overall, VCE demonstrated significant antioxidant and anti-inflammatory effects in vitro at concentrations ranging from 200 to 800 µg/mL. These concentrations were achieved dissolving a dry extract obtained from whole freeze-dried cherry waste, with ~35% extraction yield. Based on this, reaching comparable concentrations systemically would require an estimated 24–40 g of extract, equivalent to approximately 170–287 g of fresh cherries, considering their initial water content. According to the literature, a daily intake of 50 to 300 g of fresh cherries has been associated with anti-inflammatory, antioxidant, and anti-gout effects in both human and animal studies [64,76,77]. This suggests that our experimental concentration range may be achievable through regular dietary consumption, particularly with the use of concentrated preparations or optimized formulations. While such doses may be difficult to attain through diet alone, they are potentially feasible within the context of nutraceutical supplementation or pharmaceutical applications, especially when combined with bioavailability-enhancing strategies such as encapsulation, delivery systems, or co-administration with absorption enhancers. It is also important to consider that long-term or repeated intake could contribute to cumulative effects, even at lower circulating levels. Nonetheless, polyphenol bioavailability remains a significant limiting factor. Many flavonoids and phenolic acids exhibit poor gastrointestinal absorption, undergo extensive metabolism, and typically reach plasma concentrations in the nanomolar to low micromolar range, well below those used in in vitro models [78]. Furthermore, in vivo bioactivity is modulated by factors such as phase II metabolism, gut microbiota transformation, and tissue-specific distribution. Importantly, the synergistic interactions among the various bioactive constituents in VCE may enhance efficacy at lower concentrations, an effect often underestimated in single-compound studies. It should also be noted that, when suggesting daily intake levels based on fresh fruit, factors such as natural sugar content, caloric intake, and glycemic impact must be considered [79], particularly in populations with metabolic disorders. Therefore, while direct extrapolation from in vitro doses to dietary recommendations has clear limitations, our findings provide a valuable baseline for understanding the bioactivity of whole cherry waste extract and support its potential application in nutraceutical or functional food development, particularly when bioavailability challenges are addressed.

Recent studies have increasingly highlighted the anti-inflammatory and antioxidant potential of cherries and their derivatives, driven by their rich content in phenolic compounds, including flavonoids, anthocyanins, and phenolic acids. Several in vitro models using human endothelial cells have demonstrated that cherry extracts can mitigate inflammation-induced oxidative stress and endothelial dysfunction, which are key contributors to atherosclerosis and cardiovascular disease. These effects are attributed to the modulation of inflammatory markers and reactive oxygen species, although challenges related to polyphenol bioavailability remain. Nanoparticle-based delivery systems, particularly those using chitosan derivatives, have shown promise in improving cherry extract absorption and cellular uptake [55]. Other studies have evaluated cherry-derived phenolic fractions in LPS-stimulated RAW 264.7 macrophages, confirming their capacity to reduce NO levels and downregulate inflammatory enzymes such as iNOS and COX-2. Additionally, cherry fractions demonstrated selective cytotoxicity against AGS cancer cells and provided protection against oxidative stress in multiple cell lines [73]. Microwave-assisted extraction (MAE) has also been applied to enhance the recovery of bioactive compounds, resulting in extracts with increased polyphenol and anthocyanin content, as well as notable antioxidant and anti-inflammatory effects in THP-1 monocytes stimulated with monosodium urate crystals [80]. Overall, both in vitro and in vivo findings suggest that sweet cherries exhibit potent anti-inflammatory activity, partly through the inhibition of cyclooxygenase enzymes (COX-1 and COX-2), reduction of ROS, and modulation of cytokines. Their health-promoting effects may include the prevention of cardiovascular diseases, metabolic disorders, and even cancer [81].

While much of the current research focuses on specific cherry components or isolated fractions, our work presents a comprehensive characterization of a hydroethanolic extract obtained from the entire industrial cherry waste stream, including the pulp, skin, stone, and peduncle. The extract was evaluated using multiple antioxidant assays, molecular docking simulations, and in vitro biological models. However, the most innovative aspect of our study lies in the valorization of the whole cherry waste, a complex matrix usually discarded, within a circular bioeconomy framework. To the best of our knowledge, this is the first report demonstrating both the functional activity and bioactive potential of an extract derived from unfractionated whole cherry waste, highlighting a novel and sustainable approach to by-product utilization. Our study aligns with the broader context of sustainable drug discovery and bioeconomy. The use of cherry by-products not only adds value to agricultural waste but also reduces environmental impact. This is particularly relevant given the increasing emphasis on circular bioeconomy models, where waste is repurposed into valuable resources [58,60]. The hydroethanolic extract from Vignola PGI sweet cherry waste demonstrates significant antioxidant and anti-inflammatory properties. Its rich flavonoid content, coupled with its ability to modulate key inflammatory pathways such as NF-κB and MAPKs, underpins its therapeutic potential. Supported by both in vitro and in silico data, this extract represents a promising candidate for the development of nutraceuticals or adjunct therapies targeting chronic inflammation. Our study is intended as a baseline investigation, establishing a comprehensive profile of the whole sweet cherry waste extract, including both the flesh and the stone. This initial characterization serves to evaluate the feasibility of using the full extract as a functional ingredient, while setting the foundation for future studies that may focus on targeted quantification and/or bioactivity-guided fractionation. Further studies, including in vitro models of human cell lines, are needed to fully elucidate the mechanisms of action and validate its clinical relevance.

## 5. Conclusions

As we face the challenges of the modern era, this research highlights the importance of harnessing the untapped potential of agricultural by-products to build a resilient and sustainable bioeconomy. The findings of this study reveal significant antioxidant activity exhibited by a hydroethanolic extract derived from sweet cherry fruit waste. The extract showed promising anti-inflammatory effects in counteracting inflammation induced by LPS in RAW 264.7 cells. Supported by existing literature on the antioxidant and anti-inflammatory properties of *P. avium*, largely attributed to its rich phytochemical composition, particularly phenolic compounds, these results open promising avenues for the development of high-value products with therapeutic potential. The implications of this research extend beyond the laboratory, as this bioresource could serve as a basis for the development of pharmaceutical or nutraceutical products targeting inflammatory responses and oxidative stress. Future studies will aim to broaden this research by incorporating human cellular models to further explore the underlying mechanisms of the extract’s bioactivity.

## Figures and Tables

**Table 1:** TPC, TFC, and antioxidant capacity of VCE.

			Antioxidant Capacity		
	TPC (mg GAE/g)	TFC (mg QE/g)	TRP (mg AAE/g)	ABTS^+^ (mg TE/g)	DPPH (mg TE/g)
VCE	14.6 ± 1.3	24.8 ± 3.4	11.9 ± 1.1	54.3 ± 6.4	16.8 ± 1.6

Note: Data are expressed as mean ± SD (*n* = 3).

**Table 2:** Matched metabolites in *P. avium* extract.

Name	Retention Time (Min)	Formula	Calculated MW	Molecular Ion [M−H]^−^/[M+H]^+^	Mass Error (ppm)
Sakuranin	21.93	C_22_H_24_O_10_	448.1362	447.1289 [M−H]^–^	−1.69
Aequinetin	20.45	C_21_H_20_O_9_	416.1102	415.1029 [M−H]^–^	−1.32
Dihydrowogonin	21.94	C_16_H_14_O_5_	286.0837	285.0765 [M−H]^–^	−1.4
2,3-Oxiranedioctanoic acid	23.23	C_18_H_32_O_5_	328.2249	327.2176 [M−H]^–^	−0.23
Quercetin 3-rutinoside	15.32	C_27_H_30_O_16_	610.1519	609.1447 [M−H]^–^	−2.38
Quercetin	20.02	C_15_H_10_O_7_	302.0423	301.035 [M−H]^–^	−1.23
Kaempferol 3-rutinoside	11.86	C_27_H_30_O_15_	594.1574	593.1502 [M−H]^–^	−1.76
Pelargonidin	21.93	C_15_H_11_O_5_	271.0602	270.0529 [M−H]^–^	−1.68
Chrysin	27.85	C_15_H_10_O_4_	254.0577	253.0504 [M−H]^–^	−0.97
4-Ethylcatechol	22.10	C_8_H_10_O_2_	138.0677	139.075 [M+H]^+^	−2.93
4-O-p-Coumaroylquinic acid	12.91	C_16_H_18_O_8_	338.0999	337.0927 [M−H]^–^	−0.71
9,10,18-Trihydroxyoctadecanoic acid	28.69	C_18_H_36_O_5_	332.2562	331.2489 [M−H]^–^	−0.27
4-Ethylphenol	13.25	C_8_H_10_O	122.0728	123.0801 [M+H]^+^	−2.79
Naringenin 7-O-beta-D-glucoside	20.59	C_21_H_22_O_10_	434.1207	433.1134 [M−H]^–^	−1.42
(-)-Epicatechin	11.98	C_15_H_14_O_6_	290.0783	289.071 [M−H]^–^	−2.68
(+)-Naringenin	22.59	C_15_H_12_O_5_	272.0684	271.0611 [M−H]^–^	−0.26
Quercetin 3-rutinoside-4′-glucoside	13.79	C_33_H_40_O_21_	772.205	771.1978 [M−H]^–^	−1.52
Genistein 7-O-glucoside	15.49	C_21_H_20_O_10_	432.1051	431.0978 [M−H]^–^	−1.3
Oleanolic acid	44.28	C_30_H_48_O_3_	456.36	455.3526 [M−H]^–^	−0.86
Spiraeoside	15.81	C_21_H_20_O_12_	464.095	463.0877 [M−H]^–^	−1.13
Linoleic acid	46.69	C_18_H_32_O_2_	280.24	279.2327 [M−H]^–^	−0.92
p-Coumaric acid 4-O-glucoside	12.13	C_15_H_18_O_8_	326.0996	325.0923 [M−H]^–^	−1.76
Dihydroquercetin	16.21	C_15_H_12_O_7_	304.058	303.0508 [M−H]^–^	−0.91
16-Hydroxyhexadecanoic acid	44.47	C_16_H_32_O_3_	272.2351	271.2278 [M−H]^–^	−0.09
Isorhoifolin	12.54	C_27_H_30_O_14_	578.1648	579.1721 [M+H]^+^	2.23
Apigenin	22.51	C_15_H_10_O_5_	270.0526	269.0454 [M−H]^–^	−0.73
Dihydromyricetin 3-O-rhamnoside	15.07	C_21_H_22_O_12_	466.1102	465.103 [M−H]^–^	−1.89
Diosmin	12.79	C_28_H_32_O_15_	608.1727	607.1655 [M−H]^–^	−2.26
7-Hydroxysecoisolariciresinol	37.45	C_22_H_30_O_5_	374.2091	373.2018 [M−H]^–^	−0.55
3-Methylcatechol	12.54	C_7_H_8_O_2_	124.0521	125.0594 [M+H]^+^	−2.75
3-O-Feruloylquinic acid	12.11	C_17_H_20_O_9_	368.1099	367.1027 [M−H]^–^	−2.16
Oleic acid	49.53	C_18_H_34_O_2_	282.2557	281.2484 [M−H]^–^	−0.67
Biochanin A	21.39	C_16_H_12_O_5_	284.0681	283.0609 [M−H]^–^	−1.21
Malvidin 3-O-glucoside	12.84	C_23_H_25_O_12_	493.1342	492.127 [M−H]^–^	−0.73
Benzenemethanol	15.45	C_7_H_8_O	108.0575	109.0647 [M+H]^+^	−0.45
Caffeic acid 4-O-glucoside	11.70	C_15_H_18_O_9_	342.0942	341.0869 [M−H]^–^	−2.69
Palmitic acid	49.16	C_16_H_32_O_2_	256.24	255.2327 [M−H]^–^	−1.0
(Z)-beta-Damascenone	17.14	C_13_H_18_O	190.1352	191.1425 [M+H]^+^	−2.84
5-Caffeoylquinic acid	11.94	C_16_H_18_O_9_	354.0945	353.0872 [M−H]^–^	−1.66
Dihydrochrysin	21.88	C_15_H_12_O_4_	256.073	255.0657 [M−H]^–^	−2.14
Dicaffeoylquinic acid	16.61	C_25_H_24_O_12_	516.1255	515.1182 [M−H]^–^	−2.47
5-Pentadecylresorcinol	41.45	C_21_H_36_O_2_	320.2709	321.2782 [M+H]^+^	−2.0
p-Cymen-8-ol	16.47	C_10_H_14_O	150.1041	151.1113 [M+H]^+^	−2.65
Gibberellin A5	19.36	C_19_H_22_O_5_	330.1458	331.1531 [M+H]^+^	−2.71
7-O-Methylaromadendrin	22.51	C_16_H_14_O_6_	302.0788	301.0715 [M−H]^–^	−0.86
Pelargonidin 3-O-galactoside	21.94	C_21_H_21_O_10_	433.1126	432.1053 [M−H]^–^	−2.03
Dihydroquercetin 3-O-rhamnoside	17.90	C_21_H_22_O_11_	450.1156	449.1083 [M−H]^–^	−1.33
6-Methoxykaempferol	23.31	C_16_H_12_O_7_	316.0584	315.0511 [M−H]^–^	0.19
9,10-Epoxy-18-hydroxy-octadecanoic acid	32.84	C_18_H_34_O_4_	314.2458	313.2385 [M−H]^–^	0.14
Isorhamnetin 3-O-rutinoside	16.57	C_28_H_32_O_16_	624.1688	623.1616 [M−H]^–^	−0.32
Ellagic acid	15.30	C_14_H_6_O_8_	302.0058	300.9985 [M−H]^–^	−1.61
2,3-Dihydro-2,5,7-trihydroxy-2-(4-hydroxyphenyl)-4H-1-benzopyran-4-one	20.35	C_15_H_12_O_6_	288.0633	287.056 [M−H]^–^	−0.39
Kaempferol 4′-glucoside	11.65	C_21_H_20_O_11_	448.0994	447.0921 [M−H]^–^	−2.64
Kaempferol 3-rutinoside-4′-glucoside	13.92	C_33_H_40_O_20_	756.2106	755.2029 [M−H]^–^	−0.94
Cinnamic acid	33.05	C_9_H_8_O_2_	148.0523	149.0595 [M+H]^+^	−1.23
Hexadecane-1,16-dioic acid	31.97	C_16_H_30_O_4_	286.2143	285.207 [M−H]^–^	−0.33
p-Coumaric acid ethyl ester	12.66	C_11_H_12_O_3_	192.0781	193.0854 [M+H]^+^	−2.69
Malvidin	14.23	C_17_H_15_O_7_	331.0817	330.0744 [M−H]^–^	−0.21
alpha-Linolenic acid	44.12	C_18_H_30_O_2_	278.2242	277.2169 [M−H]^–^	−1.38
Octadecanoic acid	46.75	C_18_H_36_O_2_	284.2714	283.2641 [M−H]^–^	−0.54
5,7,4′-Trihydroxy-3,6-dimethoxyflavone	24.12	C_17_H_14_O_7_	330.074	329.0667 [M−H]^–^	0.11
Anethole	23.22	C_10_H_12_O	148.0885	149.0957 [M+H]^+^	−2.42
Procyanidin C1	13.74	C_45_H_38_O_18_	866.2039	865.1966 [M−H]^–^	−2.24
Eriocitrin	12.97	C_27_H_32_O_15_	596.174	595.1667 [M−H]^–^	−0.26
Naringin	20.46	C_27_H_32_O_14_	580.1785	579.1716 [M−H]^–^	−1.22
Methoxyphenylacetic acid	19.12	C_9_H_10_O_3_	166.0625	167.0698 [M+H]^+^	−2.96
Patuletin 3-O-(2″-feruloylglucosyl)(1- > 6)-[apiosyl(1- > 2)]-glucoside	13.49	C_43_H_48_O_25_	964.2466	963.2393 [M−H]^–^	−1.94
Kaempferol	22.38	C_15_H_10_O_6_	286.0477	285.0404 [M−H]^–^	−0.26
Bisdemethoxycurcumin	21.20	C_19_H_16_O_4_	308.1048	307.0975 [M−H]^–^	−0.16
Prodelphinidin dimer B3	19.13	C_30_H_26_O_14_	610.1311	609.1238 [M−H]^–^	−1.87
Isorhamnetin 3-O-galactoside	17.51	C_22_H_22_O_12_	478.111	477.1037 [M−H]^–^	−0.31
Episesamin	22.49	C_20_H_18_O_6_	354.1093	355.1166 [M+H]^+^	−2.93
Sinapaldehyde	17.65	C_11_H_12_O_4_	208.0731	207.0658 [M−H]^–^	−2.34
Sinensetin	20.39	C_20_H_20_O_7_	372.1206	371.1133 [M−H]^–^	−0.84
Lariciresinol-sesquilignan	18.95	C_30_H_36_O_10_	556.2301	555.2228 [M−H]^–^	−1.31
Conidendrin	21.57	C_20_H_20_O_6_	356.1256	355.1184 [M−H]^–^	−1.02
alpha-Ionone	17.78	C_13_H_20_O	192.1509	193.1582 [M+H]^+^	−2.46
3-Hydroxyphloretin 2′-O-glucoside	11.03	C_21_H_24_O_11_	452.1307	451.1234 [M−H]^–^	−2.59
Apigenin 7-O-glucoside	17.46	C_21_H_24_O_9_	420.1419	419.1346 [M−H]^–^	−0.41
Salicylaldehyde	26.92	C_7_H_6_O_2_	122.0367	123.0439 [M+H]^+^	−0.97
Glycitin	20.76	C_22_H_22_O_10_	446.1209	445.1133 [M−H]^–^	−0.87
Chrysoeriol 7-O-glucoside	21.63	C_22_H_22_O_11_	462.1168	461.1095 [M−H]^–^	1.22
Procyanidin B2	15.62	C_30_H_26_O_12_	578.1414	577.1341 [M−H]^–^	−1.83
Ferulic acid 4-O-glucoside	13.48	C_16_H_20_O_9_	356.1102	355.1029 [M−H]^–^	−1.63
Arctigenin	24.96	C_21_H_24_O_6_	372.1574	371.1502 [M−H]^–^	0.36
Arachidic acid	50.82	C_20_H_40_O_2_	312.3025	311.2953 [M−H]^–^	−0.95
Hesperidin	14.38	C_28_H_34_O_15_	610.1895	609.1823 [M−H]^–^	−0.4

## Data Availability

The original contributions presented in the study are included in the article; further inquiries can be directed to the corresponding author.

## Abbreviations

The following abbreviations are used in this manuscript:

VCE	Vignola Cherry Extract
TPC	Total Phenolic Content
TFC	Total Flavonoid Content
DPPH	2,2-Diphenyl-1-picrylhydrazyl
ABTS	2,2′-Azino-bis(3-ethylbenzothiazoline-6-sulfonic acid)
ROS	Reactive Oxygen Species
NO	Nitric Oxide
iNOS	Inducible Nitric Oxide Synthase
COX-2	Cyclooxygenase-2
NF-κB	Nuclear Factor kappa-light-chain-enhancer of activated B cells
MAPK	Mitogen-Activated Protein Kinase
UHPLC-ESI-MS/MS	Ultra-High Performance Liquid Chromatography–Electrospray Ionization Tandem Mass Spectrometry
MTT	3-(4,5-dimethylthiazol-2-yl)-2,5-diphenyltetrazolium bromide
DNA	Deoxyribonucleic Acid
DMEM	Dulbecco’s Modified Eagle’s Medium
DMSO	Dimethyl Sulfoxide
FBS	Fetal Bovine Serum
CCK-8	Cell Counting Kit-8
LPS	Lipopolysaccharide
DCFH_2_-DA	2′,7′-Dichlorodihydrofluorescein Diacetate
RT	Room Temperature
HRP	Horseradish Peroxidase
ECL	Enhanced Chemiluminescence
TAE	Tris-Acetate-EDTA
DAPI	4′,6-Diamidino-2-Phenylindole
PBS	Phosphate-Buffered Saline
SDS–PAGE	Sodium Dodecyl Sulfate–Polyacrylamide Gel Electrophoresis
BCA	Bicinchoninic Acid
NGS	Normal Goat Serum
TE	Trolox Equivalents

## References

[1] D’Amato D., Veijonaho S., Toppinen A. (2020). Towards Sustainability? Forest-Based Circular Bioeconomy Business Models in Finnish SMEs. For. Policy Econ..

[2] Jaouhari Y., Travaglia F., Giovannelli L., Picco A., Oz E., Oz F., Bordiga M. (2023). From Industrial Food Waste to Bioactive Ingredients: A Review on the Sustainable Management and Transformation of Plant-Derived Food Waste. Foods.

[3] Lange L., Connor K.O., Arason S., Bundgård-Jørgensen U., Canalis A., Carrez D., Gallagher J., Gøtke N., Huyghe C., Jarry B. (2021). Developing a Sustainable and Circular Bio-Based Economy in EU: By Partnering Across Sectors, Upscaling and Using New Knowledge Faster, and For the Benefit of Climate, Environment & Biodiversity, and People & Business. Front. Bioeng. Biotechnol..

[4] Morone P., Koutinas A., Gathergood N., Arshadi M., Matharu A. (2019). Food Waste: Challenges and Opportunities for Enhancing the Emerging Bio-Economy. J. Clean. Prod..

[5] Barletta R., Trezza A., Geminiani M., Frusciante L., Olmastroni T., Sannio F., Docquier J.-D., Santucci A. (2024). Chaetomorpha Linum Extract as a Source of Antimicrobial Compounds: A Circular Bioeconomy Approach. Mar. Drugs.

[6] Martini S., Conte A., Tagliazucchi D. (2019). Bioactivity and Cell Metabolism of in Vitro Digested Sweet Cherry (*Prunus avium*) Phenolic Compounds. Int. J. Food Sci. Nutr..

[7] Gençdağ E., Görgüç A., Yılmaz F.M. (2022). Valorization of Sweet Cherry (*Prunus avium*) Wastes as a Source of Advanced Bioactive Compounds. Mediterranean Fruits Bio-Wastes.

[8] Pollard Z.A., Goldfarb J.L. (2021). Valorization of Cherry Pits: Great Lakes Agro-Industrial Waste to Mediate Great Lakes Water Quality. Environ. Pollut..

[9] Boskov D., Milatovic D., Rakonjac V., Zec G., Hudina M., Veberic R., Mikulic-Petkovsek M. (2022). The Phenolic Profile of Sweet Cherry Fruits Influenced by Cultivar/Rootstock Combination. Plants.

[10] Chezanoglou E., Mourtzinos I., Goula A.M. (2024). Sweet Cherry and Its By-Products as Sources of Valuable Phenolic Compounds. Trends Food Sci. Technol..

[11] Milea A., Ștefania, Vasile A.M., Cîrciumaru A., Dumitrașcu L., Barbu V., Râpeanu G., Bahrim G.E., Stănciuc N. (2019). Valorizations of Sweet Cherries Skins Phytochemicals by Extraction, Microencapsulation and Development of Value-Added Food Products. Foods.

[12] Vignati E., Lipska M., Dunwell J.M., Caccamo M., Simkin A.J. (2022). Fruit Development in Sweet Cherry. Plants.

[13] Keane K.M., George T.W., Constantinou C.L., Brown M.A., Clifford T., Howatson G. (2016). Effects of Montmorency Tart Cherry (*Prunus cerasus* L.) Consumption on Vascular Function in Men with Early Hypertension. Am. J. Clin. Nutr..

[14] Nunes A.R., Flores-Félix J.D., Gonçalves A.C., Falcão A., Alves G., Silva L.R. (2022). Anti-Inflammatory and Antimicrobial Activities of Portuguese *Prunus avium* L. (Sweet Cherry) By-Products Extracts. Nutrients.

[15] Górnaś P., Rudzińska M., Raczyk M., Mišina I., Soliven A., Segliņa D. (2016). Composition of Bioactive Compounds in Kernel Oils Recovered from Sour Cherry (*Prunus cerasus* L.) by-Products: Impact of the Cultivar on Potential Applications. Ind. Crops Prod..

[16] Roversi A., Malvicini G.L., Porro D., Plessi C. (2010). Sweet Cherry Leaf Composition as Influenced by Genotype, Rootstock and Orchard Management. Acta Hortic..

[17] Frusciante L., Geminiani M., Olmastroni T., Mastroeni P., Trezza A., Salvini L., Lamponi S., Spiga O., Santucci A. (2024). Repurposing Castanea Sativa Spiny Burr By-Products Extract as a Potentially Effective Anti-Inflammatory Agent for Novel Future Biotechnological Applications. Life.

[18] Huang R., Wu W., Shen S., Fan J., Chang Y., Chen S., Ye X. (2018). Evaluation of Colorimetric Methods for Quantification of Citrus Flavonoids to Avoid Misuse. Anal. Methods.

[19] Frusciante L., Geminiani M., Shabab B., Olmastroni T., Roncucci N., Mastroeni P., Salvini L., Lamponi S., Trezza A., Santucci A. (2025). Enhancing Industrial Hemp (*Cannabis sativa*) Leaf By-Products: Bioactive Compounds, Anti-Inflammatory Properties, and Potential Health Applications. Int. J. Mol. Sci..

[20] Ilyasov I.R., Beloborodov V.L., Selivanova I.A., Terekhov R.P. (2020). ABTS/PP Decolorization Assay of Antioxidant Capacity Reaction Pathways. Int. J. Mol. Sci..

[21] Frusciante L., Geminiani M., Shabab B., Olmastroni T., Scavello G., Rossi M., Mastroeni P., Nyong’a C.N., Salvini L., Lamponi S. (2024). Exploring the Antioxidant and Anti-Inflammatory Potential of Saffron (*Crocus sativus*) Tepals Extract within the Circular Bioeconomy. Antioxidants.

[22] Frusciante L., Geminiani M., Trezza A., Olmastroni T., Mastroeni P., Salvini L., Lamponi S., Bernini A., Grasso D., Dreassi E. (2024). Phytochemical Composition, Anti-Inflammatory Property, and Anti-Atopic Effect of Chaetomorpha Linum Extract. Mar. Drugs.

[23] Gubitosa F., Fraternale D., Benayada L., De Bellis R., Gorassini A., Saltarelli R., Donati Zeppa S., Potenza L. (2024). Anti-Inflammatory, Antioxidant, and Genoprotective Effects of Callus Cultures Obtained from the Pulp of *Malus pumila* Cv Miller (Annurca Campana Apple). Foods.

[24] Bateman A., Martin M.J., O’Donovan C., Magrane M., Alpi E., Antunes R., Bely B., Bingley M., Bonilla C., Britto R. (2017). UniProt: The Universal Protein Knowledgebase. Nucleic Acids Res..

[25] Berman H.M., Westbrook J., Feng Z., Gilliland G., Bhat T.N., Weissig H., Shindyalov I.N., Bourne P.E. (2000). The Protein Data Bank. Nucleic Acids Res..

[26] Jumper J., Evans R., Pritzel A., Green T., Figurnov M., Ronneberger O., Tunyasuvunakool K., Bates R., Žídek A., Potapenko A. (2021). Highly Accurate Protein Structure Prediction with AlphaFold. Nature.

[27] Janson G., Paiardini A. (2021). PyMod 3: A Complete Suite for Structural Bioinformatics in PyMOL. Bioinformatics.

[28] Laskowski R.A., MacArthur M.W., Moss D.S., Thornton J.M. (1993). PROCHECK: A Program to Check the Stereochemical Quality of Protein Structures. J. Appl. Crystallogr..

[29] Stompor M. (2020). A Review on Sources and Pharmacological Aspects of Sakuranetin. Nutrients.

[30] Kim S., Chen J., Cheng T., Gindulyte A., He J., He S., Li Q., Shoemaker B.A., Thiessen P.A., Yu B. (2023). PubChem 2023 Update. Nucleic Acids Res..

[31] Carullo G., Saponara S., Ahmed A., Gorelli B., Mazzotta S., Trezza A., Gianibbi B., Campiani G., Fusi F., Aiello F. (2022). Novel Labdane Diterpenes-Based Synthetic Derivatives: Identification of a Bifunctional Vasodilator That Inhibits CaV1.2 and Stimulates KCa1.1 Channels. Mar. Drugs.

[32] Rosignoli S., Paiardini A. (2022). DockingPie: A Consensus Docking Plugin for PyMOL. Bioinformatics.

[33] Morris G.M., Huey R., Lindstrom W., Sanner M.F., Belew R.K., Goodsell D.S., Olson A.J. (2009). AutoDock4 and AutoDockTools4: Automated Docking with Selective Receptor Flexibility. J. Comput. Chem..

[34] Koebel M.R., Schmadeke G., Posner R.G., Sirimulla S. (2016). AutoDock VinaXB: Implementation of XBSF, New Empirical Halogen Bond Scoring Function, into AutoDock Vina. J. Cheminform..

[35] O’Boyle N.M., Banck M., James C.A., Morley C., Vandermeersch T., Hutchison G.R. (2011). Open Babel: An Open Chemical Toolbox. J. Cheminform..

[36] Salentin S., Schreiber S., Haupt V.J., Adasme M.F., Schroeder M. (2015). PLIP: Fully Automated Protein–Ligand Interaction Profiler. Nucleic Acids Res..

[37] Cuong N.M., Son N.T., Nhan N.T., Khanh P.N., Huong T.T., Tram N.T.T., Sgaragli G., Ahmed A., Trezza A., Spiga O. (2020). Vasorelaxing Activity of R-(−)-3′-Hydroxy-2,4,5-Trimethoxydalbergiquinol from Dalbergia Tonkinensis: Involvement of Smooth Muscle CaV1.2 Channels. Planta Med..

[38] Sievers F., Wilm A., Dineen D., Gibson T.J., Karplus K., Li W., Lopez R., McWilliam H., Remmert M., Söding J. (2011). Fast, Scalable Generation of High-quality Protein Multiple Sequence Alignments Using Clustal Omega. Mol. Syst. Biol..

[39] Sharif O., Bolshakov V.N., Raines S., Newham P., Perkins N.D. (2007). Transcriptional Profiling of the LPS Induced NF-ΚB Response in Macrophages. BMC Immunol..

[40] Shiroma Y., Fujita G., Yamamoto T., Takahashi R., Kumar A., Zhang K.Y.J., Ito A., Osada H., Yoshida M., Tahara H. (2020). Identification of a Selective RelA Inhibitor Based on DSE-FRET Screening Methods. Int. J. Mol. Sci..

[41] Scapin G., Patel S.B., Lisnock J., Becker J.W., LoGrasso P.V. (2003). The Structure of JNK3 in Complex with Small Molecule Inhibitors. Chem. Biol..

[42] Kinoshita T., Yoshida I., Nakae S., Okita K., Gouda M., Matsubara M., Yokota K., Ishiguro H., Tada T. (2008). Crystal Structure of Human Mono-Phosphorylated ERK1 at Tyr204. Biochem. Biophys. Res. Commun..

[43] Wang Z., Canagarajah B.J., Boehm J.C., Kassisà S., Cobb M.H., Young P.R., Abdel-Meguid S., Adams J.L., Goldsmith E.J. (1998). Structural Basis of Inhibitor Selectivity in MAP Kinases. Structure.

[44] Trezza A., Barletta R., Geminiani M., Frusciante L., Olmastroni T., Sannio F., Docquier J.-D., Santucci A. (2024). Chestnut Burrs as Natural Source of Antimicrobial Bioactive Compounds: A Valorization of Agri-Food Waste. Appl. Sci..

[45] Sahiner M., Yilmaz A.S., Gungor B., Ayoubi Y., Sahiner N. (2022). Therapeutic and Nutraceutical Effects of Polyphenolics from Natural Sources. Molecules.

[46] Alam W., Khan H., Shah M.A., Cauli O., Saso L. (2020). Kaempferol as a Dietary Anti-Inflammatory Agent: Current Therapeutic Standing. Molecules.

[47] Yoon J.H., Kim M.-Y., Cho J.Y. (2023). Apigenin: A Therapeutic Agent for Treatment of Skin Inflammatory Diseases and Cancer. Int. J. Mol. Sci..

[48] Nguyen M.H.T., Jung W.-K., Kim S.-K. (2011). Marine Algae Possess Therapeutic Potential for Ca-Mineralization via Osteoblastic Differentiation. Advances in Food and Nutrition Research.

[49] Trezza A., Geminiani M., Cutrera G., Dreassi E., Frusciante L., Lamponi S., Spiga O., Santucci A. (2024). A Drug Discovery Approach to a Reveal Novel Antioxidant Natural Source: The Case of Chestnut Burr Biomass. Int. J. Mol. Sci..

[50] Ginwala R., Bhavsar R., Chigbu D.I., Jain P., Khan Z.K. (2019). Potential Role of Flavonoids in Treating Chronic Inflammatory Diseases with a Special Focus on the Anti-Inflammatory Activity of Apigenin. Antioxidants.

[51] Lin Y., Bai L., Chen W., Xu S. (2010). The NF-ΚB Activation Pathways, Emerging Molecular Targets for Cancer Prevention and Therapy. Expert. Opin. Ther. Targets.

[52] Chagas M.d.S.S., Behrens M.D., Moragas-Tellis C.J., Penedo G.X.M., Silva A.R., Gonçalves-de-Albuquerque C.F. (2022). Flavonols and Flavones as Potential Anti-Inflammatory, Antioxidant, and Antibacterial Compounds. Oxidative Med. Cell. Longev..

[53] Amaro H.M., Pagels F., Tavares T.G., Costa I., Sousa-Pinto I., Guedes A.C. (2022). Antioxidant and Anti-Inflammatory Potential of Seaweed Extracts as Functional Ingredients. Hydrobiology.

[54] Jesus F., Gonçalves A.C., Alves G., Silva L.R. (2019). Exploring the Phenolic Profile, Antioxidant, Antidiabetic and Anti-Hemolytic Potential of *Prunus avium* Vegetal Parts. Food Res. Int..

[55] Beconcini D., Felice F., Fabiano A., Sarmento B., Zambito Y., Di Stefano R. (2020). Antioxidant and Anti-Inflammatory Properties of Cherry Extract: Nanosystems-Based Strategies to Improve Endothelial Function and Intestinal Absorption. Foods.

[56] Edwards M., Czank C., Woodward G.M., Cassidy A., Kay C.D. (2015). Phenolic Metabolites of Anthocyanins Modulate Mechanisms of Endothelial Function. J. Agric. Food Chem..

[57] Berni R., Cantini C., Romi M., Hausman J.-F., Guerriero G., Cai G. (2018). Agrobiotechnology Goes Wild: Ancient Local Varieties as Sources of Bioactives. Int. J. Mol. Sci..

[58] Fonseca L.R.S., Silva G.R., Luís Â., Cardoso H.J., Correia S., Vaz C.V., Duarte A.P., Socorro S. (2021). Sweet Cherries as Anti-Cancer Agents: From Bioactive Compounds to Function. Molecules.

[59] Średnicka-Tober D., Ponder A., Hallmann E., Głowacka A., Rozpara E. (2019). The Profile and Content of Polyphenols and Carotenoids in Local and Commercial Sweet Cherry Fruits (*Prunus avium* L.) and Their Antioxidant Activity In Vitro. Antioxidants.

[60] Scalbert A., Manach C., Morand C., Rémésy C., Jiménez L. (2005). Dietary Polyphenols and the Prevention of Diseases. Crit. Rev. Food Sci. Nutr..

[61] Serra A.T., Seabra I.J., Braga M.E.M., Bronze M.R., de Sousa H.C., Duarte C.M.M. (2010). Processing Cherries (*Prunus avium*) Using Supercritical Fluid Technology. Part 1: Recovery of Extract Fractions Rich in Bioactive Compounds. J. Supercrit. Fluids.

[62] Yüksekkaya Ş., Başyiğit B., Sağlam H., Pekmez H., Cansu Ü., Karaaslan A., Karaaslan M. (2021). Valorization of Fruit Processing By-Products: Free, Esterified, and Insoluble Bound Phytochemical Extraction from Cherry (*Prunus avium*) Tissues and Their Biological Activities. J. Food Meas. Charact..

[63] Bastos C., Barros L., Dueñas M., Calhelha R.C., Queiroz M.J.R.P., Santos-Buelga C., Ferreira I.C.F.R. (2015). Chemical Characterisation and Bioactive Properties of *Prunus avium* L.: The Widely Studied Fruits and the Unexplored Stems. Food Chem..

[64] Ferretti G., Bacchetti T., Belleggia A., Neri D. (2010). Cherry Antioxidants: From Farm to Table. Molecules.

[65] Fratantonio D., Cimino F., Molonia M.S., Ferrari D., Saija A., Virgili F., Speciale A. (2017). Cyanidin-3-O-Glucoside Ameliorates Palmitate-Induced Insulin Resistance by Modulating IRS-1 Phosphorylation and Release of Endothelial Derived Vasoactive Factors. Biochim. Biophys. Acta (BBA)—Mol. Cell Biol. Lipids.

[66] Xue F., Nie X., Shi J., Liu Q., Wang Z., Li X., Zhou J., Su J., Xue M., Chen W.-D. (2017). Quercetin Inhibits LPS-Induced Inflammation and Ox-LDL-Induced Lipid Deposition. Front. Pharmacol..

[67] Perkins N.D. (2007). Integrating Cell-Signalling Pathways with NF-ΚB and IKK Function. Nat. Rev. Mol. Cell Biol..

[68] Sun S.-C., Chang J.-H., Jin J. (2013). Regulation of Nuclear Factor-ΚB in Autoimmunity. Trends Immunol..

[69] Figuera-Losada M., Rojas C., Slusher B.S. (2014). Inhibition of Microglia Activation as a Phenotypic Assay in Early Drug Discovery. SLAS Discov..

[70] Ruiz P.A., Haller D. (2006). Functional Diversity of Flavonoids in the Inhibition of the Proinflammatory NF-ΚB, IRF, and Akt Signaling Pathways in Murine Intestinal Epithelial Cells. J. Nutr..

[71] Boesch-Saadatmandi C., Loboda A., Wagner A.E., Stachurska A., Jozkowicz A., Dulak J., Döring F., Wolffram S., Rimbach G. (2011). Effect of Quercetin and Its Metabolites Isorhamnetin and Quercetin-3-Glucuronide on Inflammatory Gene Expression: Role of MiR-155. J. Nutr. Biochem..

[72] Chen L., Deng H., Cui H., Fang J., Zuo Z., Deng J., Li Y., Wang X., Zhao L. (2018). Inflammatory Responses and Inflammation-Associated Diseases in Organs. Oncotarget.

[73] Gonçalves A.C., Costa A.R., Flores-Félix J.D., Falcão A., Alves G., Silva L.R. (2022). Anti-Inflammatory and Antiproliferative Properties of Sweet Cherry Phenolic-Rich Extracts. Molecules.

[74] Furman D., Campisi J., Verdin E., Carrera-Bastos P., Targ S., Franceschi C., Ferrucci L., Gilroy D.W., Fasano A., Miller G.W. (2019). Chronic Inflammation in the Etiology of Disease across the Life Span. Nat. Med..

[75] He W.-J., Lv C.-H., Chen Z., Shi M., Zeng C.-X., Hou D.-X., Qin S. (2023). The Regulatory Effect of Phytochemicals on Chronic Diseases by Targeting Nrf2-ARE Signaling Pathway. Antioxidants.

[76] Kelley D.S., Rasooly R., Jacob R.A., Kader A.A., Mackey B.E. (2006). Consumption of Bing Sweet Cherries Lowers Circulating Concentrations of Inflammation Markers in Healthy Men and Women. J. Nutr..

[77] Zhang Y., Neogi T., Chen C., Chaisson C., Hunter D.J., Choi H.K. (2012). Cherry Consumption and Decreased Risk of Recurrent Gout Attacks. Arthritis Rheum..

[78] Manach C., Williamson G., Morand C., Scalbert A., Rémésy C. (2005). Bioavailability and Bioefficacy of Polyphenols in Humans. I. Review of 97 Bioavailability Studies. Am. J. Clin. Nutr..

[79] Arbizu S., Mertens-Talcott S.U., Talcott S., Noratto G.D. (2023). Dark Sweet Cherry (*Prunus avium*) Supplementation Reduced Blood Pressure and Pro-Inflammatory Interferon Gamma (IFNγ) in Obese Adults without Affecting Lipid Profile, Glucose Levels and Liver Enzymes. Nutrients.

[80] Vírgen Gen J.J., Guzmán-Gerónimo R.I., Martínez-Flores K., Martínez-Nava G.A., Fernández-Torres J., Zamudio-Cuevas Y. (2020). Cherry Extracts Attenuate Inflammation and Oxidative Stress Triggered by Monosodium Urate Crystals in THP-1 Cells. J. Food Biochem..

[81] Faienza M.F., Corbo F., Carocci A., Catalano A., Clodoveo M.L., Grano M., Wang D.Q.-H., D’Amato G., Muraglia M., Franchini C. (2020). Novel Insights in Health-Promoting Properties of Sweet Cherries. J. Funct. Foods.

